# Case report: Kinetics of human leukocyte antigen receptor HLA-DR during liver injury induced by potassium para-aminobenzoate as assessed for causality using the updated RUCAM

**DOI:** 10.3389/fphar.2022.966910

**Published:** 2022-08-17

**Authors:** Marlene Plüß, Désirée Tampe, Harald Schwörer, Sebastian Christopher Benjamin Bremer, Björn Tampe

**Affiliations:** ^1^ Department of Nephrology and Rheumatology, University Medical Center Göttingen, Göttingen, Germany; ^2^ Department of Gastroenterology, Gastrointestinal Oncology and Endocrinology, University Medical Center Göttingen, Göttingen, Germany

**Keywords:** hepatotoxicity, acute hepatitis, drug-induced liver injury, Roussel Uclaf causality assessment method, updated RUCAM, potassium para-aminobenzoate, HLA-DR

## Abstract

Potassium para-aminobenzoate (POTABA) is used to treat Peyronie’s disease by decreasing fibrosis and plaque size progression. Among potential side effects, drug-induced liver injury (DILI) attributed to POTABA administration has been reported in a few cases and inferred to immune hypersensitivity. In the present case, we investigated clinical**,** biochemical, and serological features as well as searched for non-drug-related causes, and applied the updated Roussel Uclaf Causality Assessment Method (RUCAM) confirming a highly probable causality of POTABA-induced liver injury. Moreover, we here observed specific activated CD3^+^ T lymphocytes during the acute phase of liver injury by monitoring of human leukocyte antigen receptor (HLA-DR) expression. Furthermore, improvement of biochemical markers of liver injury after POTABA withdrawal was associated with a rapid decline of CD3^+^ HLA-DR^+^ immune cells. In contrast, CD14^+^ monocytes expressing HLA-DR remained stable during recovery from liver injury. These observations implicate a specific involvement of activated T lymphocytes in liver injury mediated by POTABA. Clinicians should be aware of POTABA-induced liver injury, and measurement of activated immune cells by assessment of HLA-DR could provide pathomechanistic insights enabling biomonitoring of recovery from DILI.

## Introduction

Drug-induced liver injury (DILI) remains a relevant problem in drug development and clinical routine. The idiosyncratic origin of DILI impedes mechanistic studies, and only little is known of its distinct pathogenesis (Cueto-Sanchez et al., 2021; Jee et al., 2021). Reactive metabolites of drugs that are bioactivated by cytochromes P450 and other enzymes in the liver are increasingly recognized to contribute to liver injury related to drug use (Chen et al., 2015). Moreover, the adaptive immune system with specific human leukocyte antigen (HLA) haplotypes is increasingly recognized as a major contributor to DILI (Stephens and Andrade, 2020). Among them, genetic HLA-DR polymorphisms have been well described and attributed to susceptibility for liver injury in the context of drug use (Nicoletti et al., 2017). However, direct assessment of HLA-DR^+^ inflammatory cells in DILI and its kinetics during liver regeneration remains elusive. Peyronie’s disease is a connective tissue disorder of the penis characterized by an abnormality in the collagen structure of the penile tunica albuginea, promoting fibrous plaque formation and fibrosis by collagen deposition (Abern et al., 2012). Peyronie’s disease can result in alterations in penile anatomy such as penile deformity, a palpable lump in the penis, penile pain during erection, and erectile dysfunction (Abern et al., 2012). Potassium para-aminobenzoate (POTABA) is a member of the vitamin B complex and used to treat Peyronie’s disease by increasing local tissue oxygenation, secretion of glycosaminoglycans, and monoamine oxidase activity to decrease fibrosis and plaque size progression (Hauck et al., 2006; Trost et al., 2007; Zhang et al., 2021). Among potential side effects, acute liver injury has been reported in a few cases and attributed to POTABA administration (Kantor and Ratz, 1985; Borum et al., 1991; Mesnil et al., 2004; Roy and Carrier, 2008; Al Attar and Kilgore, 2018). We here present kinetics of HLA-DR^+^ monocytes and T lymphocytes in POTABA-induced liver injury as assessed for causality using the updated Roussel Uclaf Causality Assessment Method (RUCAM).

## Methods

Suspected DILI can be assessed using the updated Roussel Uclaf Causality Assessment Method (RUCAM), a scoring tool determining the probability of a causal link between a noxious agent (a drug or herb) and liver injury (Danan and Teschke, 2015; Danan and Teschke, 2019). Updated RUCAM is a standardized and validated method specific to hepatotoxicity and has long been well-established in hepatological practice, studies, registries, and case reports (Benichou et al., 1993; Danan and Benichou, 1993). The expression of HLA-DR on CD14^+^ monocytes and CD3^+^ T lymphocytes was analyzed using flow cytometry, as previously described (Dickel et al., 2021; Grimm et al., 2021; Baier et al., 2022; Tampe et al., 2022). Antibody staining followed standard protocols for antibody staining of cells in suspension. Within 1 h after collection, cells were incubated with antibodies against HLA-DR for 15 min at 4°C in the dark and gated according to positive and isotype negative controls.

## Case description

**FIGURE 1:**
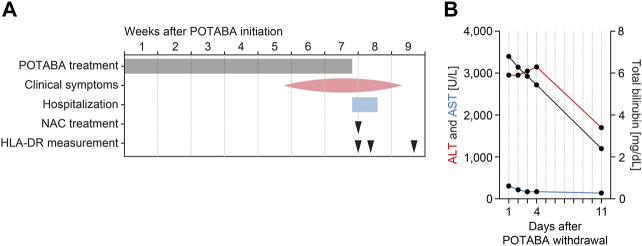
Timeline of the case. **(A)** Time course relative to initiation of POTABA treatment. **(B)** Levels of transaminases (ALT and AST, plotted to the left axis) and total bilirubin levels (plotted to the right axis) after withdrawal of POTABA. Abbreviations: ALT, alanine transaminase; AST, aspartate aminotransferase; HLA-DR, human leukocyte antigen receptor; NAC, N-acetyl cysteine; POTABA, potassium para-aminobenzoate.

A 73-year-old man presented to our emergency department with a history of dark yellow urine for 2 weeks, as well as jaundice for 3 days (Figure 1A). The patient had a prior history of Peyronie’s disease treated with POTABA for 7 weeks before admission, no other medication was present (Figure 1A). On admission, there was no fever, hepatomegaly, or abdominal tenderness. The patient denied a history of alcohol consumption or drug abuse, without previous elevated liver enzymes. A history of recent acute hypotension was denied and vital parameters at admission were stable. A nasopharyngeal swab for SARS-CoV-2 RNA testing was negative. Laboratory examination revealed elevated transaminases (ALT: 3,399 U/L, normal range: ≤45 U/L; AST: 304 U/L, normal range: ≤35 U/L), and total bilirubin levels (5.9 mg/dl, normal range: 0.3–1.2 mg/dl, Table 1). A hepatobiliary ultrasound including colour doppler sonography of liver vessels did not show any pathological findings. A subsequent in-depth diagnostic workup excluded infectious (leptospirosis, viral hepatitis) or autoimmune causes of acute liver injury (Table 1). Applying RUCAM to the present case revealed that the type of liver injury was classified as hepatocellular injury as opposed to cholestatic or mixed liver injury by the ratio of ALT over alkaline phosphatase (AP, ratio: >5). By using the updated RUCAM for hepatocellular injury, a total score of 10 points and hence a causality grading of ‘highly probable’ was determined for the present case (Table 2) (Danan and Teschke, 2015). This strongly supported our conclusion that the liver injury in this case was caused by POTABA. For supportive therapy, POTABA treatment was withdrawn and N-acetyl cysteine (NAC) as well as intravenous rehydration was initiated (Figure 1A). Thereafter, biochemical markers of liver injury improved, and the patient was discharged 4 days after admission. Clinical jaundice further resolved until an ambulatory follow-up 10 days after discharge, associated with further improvement of transaminases (ALT: 1,197 U/L, normal range: ≤45 U/L; AST: 139 U/L, normal range: ≤35 U/L), and total bilirubin levels (3.4 mg/dl, normal range: 0.3–1.2 mg/dl, Figure 1A).

**TABLE 1:** Laboratory parameters at admission.

Parameter	Value	Normal range
Hemoglobin—g/dL	14.5	13.5-17.5
Leukocytes—1,000/µL	4.26	4-11
INR—ratio	1.4	0.8-1.2
aPTT—seconds	29	25-37
AST—U/L	304	≤35
ALT—U/L	3,399	≤45
AP—U/L	280	40-150
GGT—U/L	264	12-64
CHE—U/L	5,132	4,389-10,928
Lipase—U/L	43	<60
Haptoglobin—g/L	0.41	0.14-2.58
LDH—U/L	660	125-250
Total bilirubin—mg/dL	5.9	0.3-1.2
Conjugated bilirubin—mg/dL	4.6	≤0.5
Creatinine—mg/dL	0.9	0.7-1.2
Circulating immune complexes—µg/mL	40.1	<45
AMA-M2—IU/mL	0.6	<1
IgG 4—g/L	0.216	0.03-2.01
ANCA-IF—titer	Neg	Neg
PR3-ANCA—IU/mL	0.2	<2
MPO-ANCA—IU/mL	0.2	<3.5
ENA screen	0.1	<0.7
ANA-IF—titer	1:100	<1:100
AMA-IF—titer	Neg	Neg
ASMA-IF—titer	Neg	Neg
*Leptospira* ELISA—DU	7.45	<9
EBV-DNA—copies/mL	Neg	Neg
CMV-DNA—IU/mL	<35	<35
Anti-HAV	Pos	Neg
Anti-HAV IgM	Neg	Neg
Hbs antigen	Neg	Neg
Anti-HBc	Neg	Neg
Anti-HBs—mIU/mL	Neg	Neg
Anti-HCV	Neg	Neg
Anti-HEV IgG	Neg	Neg
Anti-HEV IgM	Neg	Neg
IgA—g/L	3.14	0.63-4.85
IgG—g/L	12.9	5.4-18.2
IgM—g/L	1.23	0.22-2.93

Abbreviations: AMA, anti-mitochondrial antibody; AMA-M2, anti-mitochondrial M2 antibody; ANA, antinuclear antibody; ANCA, antineutrophil cytoplasmic antibody; ALT, alanine transaminase; AP, alkaline phosphatase; aPTT, activated partial thromboplastin time; ASMA, anti-smooth muscle antibody; AST, aspartate aminotransferase; CHE, cholinesterase; CMV, cytomegalovirus; EBV, Epstein-Barr virus; ELISA, enzyme-linked immunoassay; ENA, extractable nuclear antigen; GGT, gamma-glutamyl transferase; HAV, hepatitis A virus; HCV, hepatitis C virus; HEV, hepatitis E virus; HBc, hepatitis B core antigen; HBs, hepatitis B surface antigen; IgA, immunoglobulin A; IF, immunofluorescence; IgG, immunoglobulin G; IgG 4, immunoglobulin G 4; IgM, immunoglobulin M; INR, international normalized ratio; LDH, lactate dehydrogenase; MPO-ANCA, myeloperoxidase-ANCA; Neg, negative; PR3-ANCA, proteinase 3-ANCA.

**TABLE 2:** Updated RUCAM for the hepatocellular injury of DILI.

Items for hepatocellular injury	Score
1. Time to onset from the beginning of the drug: 5–90 days	+2
2. Course of ALT after cessation of the drug: Decrease ≥50% within 8 days	+3
3. Risk factors: Age ≥55 years	+1
4. Concomitant drug(s): None or no information	0
5. Search for alternative causes: All causes-groups I and II reasonably ruled out	+2
6. Previous hepatotoxicity of the drug: Reaction labelled in the product characteristics	+2
7. Response to unintentional re-exposure: Other situations	0

Abbreviations: ALT, alanine transaminase; DILI, drug-induced liver injury; RUCAM, Roussel Uclaf Causality Assessment Method.

## Kinetics of HLA-DR^+^ leukocytes

**FIGURE 2:**
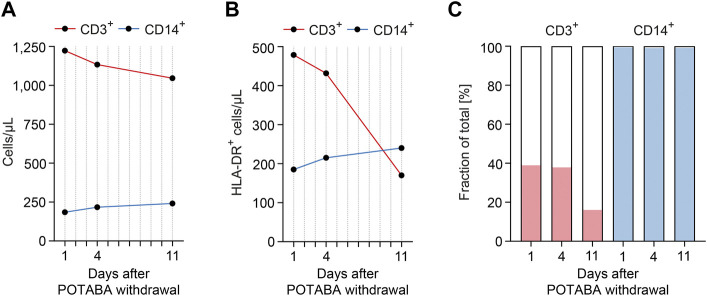
Kinetics of HLA-DR+ T lymphocytes associate with recovery from hepatotoxicity induced by POTABA. **(A,B)** Monitoring of circulating total and HLA-DR^+^ monocytes (CD14^+^ HLA-DR^+^) and T lymphocytes (CD3^+^ HLA-DR^+^) by flow cytometry revealed that improvement of liver injury after POTABA withdrawal resulted in a rapid decline of absolute CD3^+^ HLA-DR^+^ T lymphocyte counts, while CD14^+^ HLA-DR^+^ monocytes remained unaffected. **(C)** Fraction of HLA-DR^+^ cells within the total monocyte (CD14^+^) and T lymphocyte (CD3^+^) populations confirm that improvement of liver injury after POTABA withdrawal resulted in a rapid decline of relative CD3^+^ HLA-DR^+^ T lymphocyte counts. Abbreviations: HLA-DR, human leukocyte antigen receptor; POTABA, potassium para-aminobenzoate.

A differential blood count at admission showed normal distribution of lymphocytes with only decreased B lymphocytes (36 cells/µL, normal range: 100-500 cells/µL, Table 3). Flow cytometry measurements of circulating activated monocytes and T lymphocytes reflected by cell surface HLA-DR expression revealed presence of HLA-DR on the surface of monocytes (CD14^+^ HLA-DR^+^: 185 cells/µL, 99.7% of the CD14^+^ population) and T lymphocytes (CD3^+^ HLA-DR^+^: 479 cells/µL, 39.2% of the CD3^+^ population, Figures 2A,B; Table 3). Monitoring of HLA-DR revealed that improvement of liver injury after POTABA withdrawal resulted in a rapid decline of absolute and relative CD3^+^ HLA-DR^+^ T lymphocyte counts within the total population (Figures 2A–C; Table 3). Contrasting to this observation, absolute and relative counts of CD14^+^ HLA-DR^+^ monocytes remained unaffected (Figures 2A–C; Table 3). In summary, activated CD3^+^ T lymphocytes expressing HLA-DR are present during the acute phase of liver injury. Furthermore, improvement of biochemical markers of liver injury after POTABA withdrawal was associated with a rapid decline of CD3^+^ HLA-DR^+^ immune cells.

**TABLE 3:** HLA-DR flow-cytometry results relative to POTABA withdrawal.

Parameter	Marker	Day 1	Day 4	Day 11
Th cells—cells/µL	CD3^+^/CD4^+^	511		
NK cells—cells/µL	CD3^-^/CD56^+^/CD16^+^	103		
B cells—cells/µL	CD19^+^	36		
T suppressor cells– cells/µL	CD3^+^/CD14^+^	813		
Monocytes—cells/µL	CD14^+^	185	217	241
HLA-DR^+^—cells/µL (%)	CD14^+^/HLA-DR^+^	185 (99.7)	215 (99.3)	240 (99.4)
T lymphocytes—cells/µL	CD3^+^	1,223	1,134	1,046
HLA-DR^+^—cells/µL (%)	CD3^+^/HLA-DR^+^	479 (39.2)	432 (38.1)	170 (16.3)

Abbreviations: HLA-DR, human leukocyte antigen receptor; POTABA, potassium para-aminobenzoate; Th, T helper; NK, natural killer.

## Follow-up monitoring and outcome

An ambulatory follow-up visit 88 days after POTABA withdrawal confirmed full resolution of clinical jaundice and biochemical markers of liver injury (ALT: 26 U/L, normal range: ≤45 U/L; AST: 39 U/L, normal range: ≤35 U/L; total bilirubin levels: 0.9 mg/dl, normal range: 0.3–1.2 mg/dl, Table 4). In addition, no seroconversion of autoimmune markers following the initial liver damage was detectable (Table 4). These observations further confirm a highly probable causality for POTABA-induced liver injury. In addition, low levels of absolute and relative CD3^+^ HLA-DR^+^ T lymphocyte counts within the total population remained stable (Table 5). The patient will be closely monitored for signs of liver injury, and continued POTABA avoidance was recommended.

**TABLE 4:** Laboratory parameters at a follow-up visit 88 days after POTABA withdrawal.

Parameter	Value	Normal range
Hemoglobin—g/dL	15.8	13.5-17.5
Leukocytes—1,000/µL	4.08	4-11
INR—ratio	1.2	0.8-1.2
AST—U/L	39	≤35
ALT—U/L	26	≤45
AP—U/L	82	40-150
GGT—U/L	32	12-64
LDH—U/L	123	125-250
Total bilirubin—mg/dL	0.9	0.3-1.2
Conjugated bilirubin—mg/dL	0.4	≤0.5
Creatinine—mg/dL	0.98	0.7-1.2
AMA-M2—IU/mL	0.5	<1
Anti-M2-3E (BPO)—blot	Neg	Neg
Anti-LKM-1—blot	Neg	Neg
Anti-LC-1—blot	Neg	Neg
Anti-SLA/LP—blot	Neg	Neg
ANCA-IF—titer	Neg	Neg
PR3-ANCA—IU/mL	<0.2	<2
MPO-ANCA—IU/mL	<0.2	<3.5
ENA screen	0.1	<0.7
ANA-IF—titer	1:100	<1:100
AMA-IF—titer	Neg	Neg
IgA—g/L	4	0.63-4.85
IgG—g/L	16	5.4-18.2
IgM—g/L	1.48	0.22-2.93

Abbreviations: AMA, anti-mitochondrial antibody; AMA-M2, anti-mitochondrial M2 antibody; ANA, antinuclear antibody; ANCA, antineutrophil cytoplasmic antibody; ALT, alanine transaminase; AP, alkaline phosphatase; AST, aspartate aminotransferase; ENA, extractable nuclear antigen; GGT, gamma-glutamyl transferase; IgA, immunoglobulin A; IF, immunofluorescence; IgG, immunoglobulin G; IgM, immunoglobulin M; INR, international normalized ratio; LDH, lactate dehydrogenase; LC-1, liver cytosol antibody type 1; LKM-1, liver/kidney microsome type 1; MPO-ANCA, myeloperoxidase-ANCA; Neg, negative; PR3-ANCA, proteinase 3-ANCA; SLA/LP, soluble liver antigen/liver pancreas.

**TABLE 5:** HLA-DR flow-cytometry results at a follow-up visit 88 days after POTABA withdrawal.

Parameter	Marker	Value
Monocytes—cells/µL	CD14^+^	162
HLA-DR^+^—cells/µL (%)	CD14^+^/HLA-DR^+^	161 (99.5)
T lymphocytes—cells/µL	CD3^+^	962
HLA-DR^+^—cells/µL (%)	CD3^+^/HLA-DR^+^	155 (16.1)

Abbreviations: HLA-DR, human leukocyte antigen receptor; POTABA, potassium para-aminobenzoate.

## Discussion

Hepatotoxicity and liver injury associated with POTABA intake was first reported in 1985, a rare side effect reported in only 7 cases as of yet (Kantor and Ratz, 1985; Borum et al., 1991; Mesnil et al., 2004; Roy and Carrier, 2008; Al Attar and Kilgore, 2018). In line with the present case, liver injury occurred 4–8 weeks after initiation of POTABA treatment (Roy and Carrier, 2008; Al Attar and Kilgore, 2018). In addition, treatment with N-acetyl cysteine has also been considered in one past case (Roy and Carrier, 2008). Therefore, no further investigation including a liver biopsy during the acute phase of illness was required. Also, a common finding between the present and reported cases was a resolution of liver injury markers after POTABA withdrawal (Roy and Carrier, 2008; Al Attar and Kilgore, 2018). As such, it was inferred that liver injury was due to immune hypersensitivity to POTABA.

Hepatotoxicity and liver injury related to drug use are rare but important complications, because these can potentially cause acute liver failure leading to death or requirement of liver transplantation. In clinical practice, assessment of causality in the context of DILI is flawed by alternative diagnoses, as these patients are not provided in time with the appropriate specific therapies substantially different from those of the initial incorrect diagnosis of DILI (Aithal et al., 1999; Dalton et al., 2007; Davern et al., 2011; Teschke et al., 2013; Teschke et al., 2014; Teschke et al., 2016). Missed diagnoses are often described in the literature and could occur at any evaluating level, beginning with the caring physician, continuing among expert groups, and ending during the evaluation by the regulatory agencies (Teschke et al., 2013; Teschke et al., 2014). These specific problems among other confounders like poor data quality, comedication, and inconsistent interpretation of drug challenge, withdrawal and re-challenge in DILI have early been recognized and led to the development of a causality assessment method named RUCAM (Benichou et al., 1993; Danan and Benichou, 1993). Updated RUCAM includes clinical**,** biochemical, and serological features as well as searched for non-drug causes to assess causality in suspected DILI (Benichou et al., 1993; Danan and Benichou, 1993; Danan and Teschke, 2015; Danan and Teschke, 2019). POTABA-induced liver injury has been described previously, while verified RUCAM-based causality was not confirmed in all these cases (Table 4). Furthermore, recalculation of the updated RUCAM based on the published information revealed incomplete investigation of especially alternative causes among these cases of suspected POTABA-induced liver injury (Table 6) (Benichou et al., 1993; Danan and Benichou, 1993; Danan and Teschke, 2015; Danan and Teschke, 2019). Based on these observations, assessment of the updated RUCAM should be recommended to evaluate probability of causality in suspected DILI.

**TABLE 6:** Published cases of POTABA-induced liver injury.

References	No. of cases	RUCAM included	Updated RUCAM (recalculated)	Causality grading
Kantor and Ratz (1985)	1	No	5	Possible
Borum et al. (1991)	1	No	5	Possible
Mesnil et al. (2004)	3	No	5	Possible
Roy and Carrier (2008)	1	No	5	Possible
Al Attar and Kilgore (2018)	1	No	5	Possible

Abbreviations: No., number, POTABA, potassium para-aminobenzoate; RUCAM, Roussel Uclaf Causality Assessment Method.

The development of DILI is considered to be multifactorial, depending on a combination of drug properties, immunological determinants, as well as environmental factors (Chen et al., 2015). The immune system is believed to play a fundamental role in DILI development and progression (Cueto-Sanchez et al., 2021; Jee et al., 2021). This is supported by findings of specific HLA alleles associated with DILI susceptibility to specific drugs (Stephens and Andrade, 2020). Among them, genetic HLA-DR polymorphisms have been well described and attributed to susceptibility for liver injury in the context of drug use (Nicoletti et al., 2017). Metabolites resulting from drug metabolism may act as haptens and bind to endogenous proteins to form adducts that may trigger T-cell activation and an immune response when presented on specific HLA-DR molecules. However, the liver is constantly exposed to xenoantigens requiring immune tolerance for protection from autoinflammation (Dara et al., 2016). Therefore, idiosyncratic DILI is believed to result from a disrupted tolerogenic state (Dara et al., 2016). In fact, multiple liver diseases have been associated with alterations in immune tolerance, such as autoimmune hepatitis, primary biliary cirrhosis, and primary sclerosing cholangitis (Chung and Hirschfield, 2017; Czaja, 2018; Tanaka et al., 2019). For the first time, we here observed activated CD3^+^ T lymphocytes reflected by presence of HLA-DR during the acute phase of liver injury. Furthermore, improvement of liver injury biochemical markers after POTABA withdrawal was associated with a rapid decline of CD3^+^ HLA-DR^+^ immune cells. In contrast, CD14^+^ monocytes expressing HLA-DR remained stable during recovery from liver injury. Our observations implicate a specific involvement of activated CD3^+^ HLA-DR^+^ T lymphocytes in POTABA-induced liver injury. In conjunction with the CD3/T cell receptor (TCR) complex and CD4 molecules, HLA-DR expression reflects T lymphocyte activation and is critical for efficient peptide presentation to CD4^+^ T lymphocytes (Unanue et al., 1984). The causal contribution of HLA-DR^+^ immune cells to acute liver injury has already been experimentally described, requiring further investigation (Beringer et al., 2019). Although most of the liver injury is likely mediated by the adaptive immune system, its activation requires an innate immune response to activate antigen presenting cells and produce cytokines required for T cell proliferation. Therefore, we here provide first evidence for an involvement of activated CD3^+^ HLA-DR^+^ T lymphocytes in DILI, particularly to POTABA therapy. We are aware that this observation is not causal and attributable to every drug and DILI. However, we here provide first evidence that HLA-DR^+^ immune cells may allow biomonitoring of liver injury and recovery from DILI. In the context of POTABA, clinicians should be aware of POTABA-induced liver injury, and the correct management appears to be the immediate withdrawal of POTABA and supportive therapy. Finally, measurement of activated immune cells by assessment of HLA-DR could provide pathomechanistic insights into idiosyncratic DILI.

## Data Availability

The original contributions presented in the study are included in the article/supplementary material, further inquiries can be directed to the corresponding author.
